# Virtual patient identifier (vPID): Improving patient traceability using anonymized identifiers in Japanese healthcare insurance claims database

**DOI:** 10.1016/j.heliyon.2023.e16209

**Published:** 2023-05-12

**Authors:** Jumpei Sato, Naohiro Mitsutake, Hiroyuki Yamada, Masaru Kitsuregawa, Kazuo Goda

**Affiliations:** a Institute of Industrial Science, The University of Tokyo, Meguro-ku, Tokyo, Japan; b Institute for Health Economics and Policy, Minato-ku, Tokyo, Japan

**Keywords:** Personal identifier, Healthcare insurance claims database, Patient traceability

## Abstract

**Objective:**

Japan's national-level healthcare insurance claims database (NDB) is a collective database that contains the entire information on healthcare services being provided to all citizens. However, existing anonymized identifiers (ID1 and ID2) have a poor capability of tracing patients' claims in the database, hindering longitudinal analyses. This study presents a virtual patient identifier (vPID), which we have developed on top of these existing identifiers, to improve the patient traceability.

**Methods:**

vPID is a new composite identifier that intensively consolidates ID1 and ID2 co-occurring in an identical claim to allow to collect claims of each patient even though its ID1 or ID2 may change due to life events or clerical errors. We conducted a verification test with prefecture-level datasets of healthcare insurance claims and enrollee history records, which allowed us to compare vPID with the ground truth, in terms of an identifiability score (indicating a capability of distinguishing a patient's claims from another patient's claims) and a traceability score (indicating a capability of collecting claims of an identical patient).

**Results:**

The verification test has clarified that vPID offers significantly higher traceability scores (0.994, Mie; 0.997, Gifu) than ID1 (0.863, Mie; 0.884, Gifu) and ID2 (0.602, Mie; 0.839, Gifu), and comparable (0.996, Mie) and lower (0.979, Gifu) identifiability scores.

**Discussion:**

vPID is seemingly useful for a wide spectrum of analytic studies unless they focus on sensitive cases to the design limitation of vPID, such as patients experiencing marriage and job change, simultaneously, and same-sex twin children.

**Conclusion:**

vPID successfully improves patient traceability, providing an opportunity for longitudinal analyses that used to be practically impossible for NDB. Further exploration is also necessary, in particular, for mitigating identification errors.

## 1 Introduction

Japan is the world's fastest aging society [[1], [2], [3]]. The population ratio of elderly citizens (i.e., aged 65 and above) was 9.10% in 1980, but it drastically grew up to 23.0% in 2010, and it is projected to reach 36.1% by 2040 [3,4]. Accordingly, the medical expenditure has been steadily increasing, whereas the economic growth has remained sluggish. The national healthcare spending accounted for only 6.25% of the gross domestic product in 1980, but it sharply rose up to 9.18% in 2010 [5]. Japan has been struggling to keep providing high-quality healthcare services while controlling the rising expenditure.

One promising approach is data-oriented analyses to understand the reality of healthcare service provision and to induce the reform of the national healthcare system. In April 2009, the Ministry of Health, Labour and Welfare (MHLW) of Japan started collecting all healthcare insurance claims data from all public healthcare insurers to build the *National Database of Health Insurance Claims and Specific Health Checkups of Japan* (NDB) [[6], [7], [8]]. Japan has employed the universal healthcare insurance policy since 1961 [9,10]; all the Japanese citizens and qualified non-Japanese residents are forced to enroll in any public healthcare insurance programs, which cover all of the necessary and approved healthcare services. Each insurance claim records descriptive information of a patient, a healthcare service provider (e.g., hospital), an involved insurance program, medical diagnoses, healthcare services (e.g., drug prescriptions and medical treatments), and a claimed expense in a standardized electronic format [8]. Potentially, NDB contains the entire information about healthcare services being provided to the citizens. The database (more than 14,810 million claims of 126 million citizens as of March 2018 [11]) has been actively studied [[12], [13], [14], [15], [16], [17], [18], [19], [20], [21], [22], [23], [24], [25], [26]].

A drawback of NDB is its poor capability of longitudinally tracing patients' claims. NDB does not hold original personal identification information for privacy protection; instead, it contains two anonymized identifiers, ID1 and ID2, which are generated from patient information, and are thus subject to life events or clerical errors. The naïve use of ID1 and ID2 often fails to trace all the claims correctly for every patient. Thoroughly reviewing a patient's history of healthcare services is an essential part of longitudinal analyses [[27], [28], [29]]. This limited capability poses an impediment for such analyses on NDB.

We have developed a new composite identifier, named *virtual patient identifier* (vPID), on top of ID1 and ID2 on NDB [12]. vPID is being actually deployed to healthcare study projects [15,17,18]. The basic idea is to intensively consolidate ID1 and ID2 co-occurring in an identical claim in order to improve the patient traceability. This paper presents a verification test that we conducted with the datasets provided by 219 regional public healthcare insurers. These datasets contained healthcare insurance claims and enrollee history records, which allowed for direct comparison with the ground truth. The vPID solution assumes that only either of ID1 and ID2 changes at the same time. Our verification has demonstrated that this assumption is practically accepted; vPID significantly improves the patient traceability with small identification errors.

Obviously, it would be desirable for the patient database to hold a perfectly accurate identifier. A possible solution is to rebuild NDB with the revised anonymous identifier design from scratch. Alternatively, how to exploit the currently available (partially flawed) dataset seems worthy of exploration; even though the database has an imperfect identifier, it has accumulated the national-level dataset for more than ten years, helping us to capture novel knowledge. We initially developed vPID to overcome the poor traceability of NDB. The same idea can be potentially applied to other scenarios, such as analyzing a patient database or another population database that lacks a perfectly accurate identifier but only holds multiple anonymized identifiers. This paper would help those who need to utilize a patient database with similar identifier problems.

## 2 Materials and methods

### 2.1 Problem definition

In NDB, personal identification information (e.g., patient's insured identifier and name) is removed from each insurance claim for privacy protection, and instead, the following two anonymized identifiers are imparted thereto.

‧ ID1: a hash value generated from a patient's insured identifier, gender, and birthdate; and
‧ ID2: a hash value generated from a patient's name, gender, and birthdate.

The insured identifier is a unique number assigned to every insured family or citizen at the national level. Analysts are supposed to use these anonymized identifiers for their analysis work. However, these identifiers have a poor capability to trace the medical history of each patient because they may change with life events or clerical errors. For example, when a citizen changes its workplaces, its healthcare insurance transfers from the former occupational insurance program to a new one; its ID1 is likely to change. When a couple gets married, either of the couple changes its family names in Japan's marriage system; its ID2 is likely to change. Furthermore, clerical errors such as typos also induce the unexpected change of identifiers; some *kanji* characters (used for spelling Japanese names) have multiple font styles, being likely to induce clerical errors. Our study indicates that ID1 and ID2 correctly trace all the insurance claims only for 60.2%–88.4% of patients, as presented in Section 3.

### 2.2 Virtual patient identifier (vPID)

**Fig. 1:**
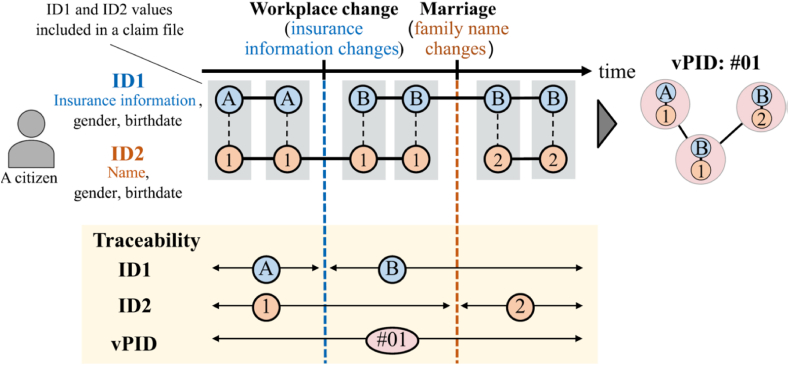
An idea of virtual patient identifier (vPID).

We have developed a new identifier, named *virtual patient identifier* (vPID), to improve the patient traceability for NDB. Fig. 1 illustrates the approach of vPID. The existing identifiers are subject to life events and clerical errors as described in the previous section. The naïve use of ID1 or ID2 does not trace healthcare insurance claims beyond such life events and clerical errors.

Our solution is to intensively merge ID1 and ID2 that occur in identical claims and give a unique vPID value for each merged set of ID1 and ID2. The current implementation sorts ID1 and ID2 values that belong to each vPID instance and then calculates a concatenated value of them to obtain a vPID value. Intensive identifier consolidation potentially allows us to connect claims beyond life events and clerical errors, helping improve the capability of tracing patients’ claims. The vPID generation algorithm is formally described in Supplementary Section S1. vPID assumes that even if ID1 changes, ID2 is unlikely to change at the same time, and vice versa. If ID1 and ID2 change for a certain patient at the same time, even vPID is not able to trace its claims beyond the change. Our verification test presented later has confirmed that this assumption is practically acceptable, and vPID improves the patient traceability significantly with small identification errors, although vPID is not a perfect solution.

### 2.3 Validation method and indicators

We comparatively validated the accuracy of ID1, ID2, and vPID. We obtained ground truth by combining healthcare insurance claims and enrollment history records. For a given anonymized identifier, the enrollment history records offer anonymized unique citizen numbers, which are reliable identifiers for correctly distinguishing unique patients.

We defined the following two accuracy indicators. Roughly speaking, the identifiability score indicates the ratio of correctly distinguishing every patient's claims from the other patients' claims, whereas the traceability score indicates the ratio of collecting all the claims of every patient. Their formal definition is presented as follows. Both scores take values between 0 and 1; greater values indicate higher accuracy. Identifiability and traceability are more likely to present lower scores than precision and recall in the classification problem, even though they are semantically analogous. Their formal definition is presented in Supplementary Section S2.

### 2.4 Test datasets

For validation, the present study has utilized prefecture-level datasets (Mie and Gifu datasets), which have been provided by 219 regional public healthcare insurers (covering self-employed workers and retired/non-working citizens in all the local government territories in the Mie and Gifu prefectures). Table 1 summarizes the dataset statistics. The Mie dataset contains 71,533 thousand healthcare insurance claims and 2699 thousand enrollment history records of 837 thousand insured citizens from March 2013 through November 2017, whereas the Gifu dataset contains 74,346 thousand claims and 4098 thousand records of 1010 thousand insured citizens from April 2014 through March 2018. Moreover, this study demonstrates the practical effectiveness of vPID by utilizing the NDB dataset, which has been provided by MHLW [30]. Table 1 summarizes the dataset statistics as well. The NDB dataset contains 9464 million healthcare insurance claims, which cover most of the claims stored in the original NDB from April 2009 through March 2015.

**Table 1:** Dataset statistics.

	Mie dataset	Gifu dataset	NDB dataset
Data provider	91 regional public insurers covering 29 local governments in the Mie prefectural area	128 regional public insurers covering 42 local governments in the Gifu prefectural area	All the public insurers covering all 47 prefectural areas
Data period	March 2013 through November 2017	April 2014 through March 2018	April 2009 through March 2015
Healthcare insurance claims	71,533 thousand claims	74,346 thousand claims	9464 million claims
Enrollment history records	2699 thousand records	4098 thousand records	N/A
Insured citizens	837 thousand citizens	1010 thousand citizens	N/A

## 3 Results

### 3.1 Validation of the accuracy of virtual patient identifier (vPID)

First, we present an experimental result on the accuracy of the identifiers. Table 2 summarizes the result, which suggests that vPID offers significantly higher traceability than ID1 and ID2 in the Mie and Gifu datasets.

**Table 2:** Validation result of different identifiers using prefecture-level claims datasets.

	Mie dataset			Gifu dataset		
	ID1	ID2	vPID	ID1	ID2	vPID
**Number of instances (thousand)**	1106	1449	937	1190	1479	1016
**Identifiability score**	0.999	0.999	0.996	0.999	0.997	0.979
**Traceability score**	0.863	0.602	0.994	0.884	0.839	0.997
**Average number of instances per an insured citizen**	1.206	1.584	1.006	1.191	1.473	1.003

The Mie and Gifu datasets held cardinalities (i.e., numbers of different values) of 1106 thousand and 1190 thousand for ID1, respectively, which ID1 offered an identifiability score of 0.999 but traceability scores of 0.863–0.884. This result indicates that ID1 can almost perfectly distinguish a patient's claims from another patient's claims but fails to collect all the claims for more than 11.6% of the patients. As noted above, ID1 was generated from a patient's insured identifier, gender, and birthdate. The observed unsuccessful cases were seemingly due to life events and clerical errors, which induced the change of ID1s. On average, a single insured citizen in the Mie and Gifu was given 1.206 and 1.191 different ID1 values, respectively, during the test period. ID2 also presented a similar result. We confirmed cardinalities of 1449 thousand and 1479 thousand for ID2 in the Mie and Gifu datasets, respectively, wherein ID2 offered identifiability scores of 0.997–0.999 but traceability scores of 0.602–0.839. This result indicates that ID2 also has almost perfect identifiability but fails in claims tracing for a substantial population of patients. Furthermore, ID2 (being generated from a patient's name, gender, and birthdate) was also subject to life events and clerical errors, which seemed to cause the failed cases. In particular, the name element seemed to degrade the traceability of ID2 since names are likely to induce clerical errors. On average, a single insured citizen in the Mie and Gifu was given 1.584 and 1.473 different ID2 values.

In contrast, vPID achieved much higher traceability scores of 0.994 (Mie) and 0.997 (Gifu), and a comparably high identifiability score of 0.996 (Mie) and a lower score of 0.979 (Gifu). The proposed algorithm intensively merged patient identifiers on the basis of ID1 and ID2 beyond life events and clerical errors. Actually, we confirmed cardinalities of only 937 thousand and 1016 thousand for vPID in the Mie and Gifu datasets, respectively. On average, a single insured citizen in Mie and Gifu was given 1.006 and 1.003 different vPID values, respectively, for the test period. Only 4875 (0.582%) and 3221 (0.319%) insured citizens were given multiple vPID values due to the simultaneous change of ID1 and ID2. That is how vPID had significantly higher traceability in comparison with ID1 and ID2, even though it yielded a certain amount of identification errors.

### 3.2 Identifier consolidation effect of the vPID algorithm

**Fig. 2:**
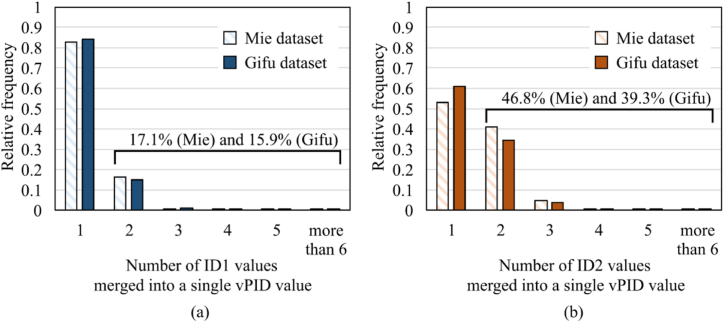
Fraction histograms of the numbers of ID1 and ID2 values merged into a single vPID value. (a) ID1, (b) ID2.

Here, we show how ID1 and ID2 are merged by the vPID algorithm. Fig. 2 indicates that vPID intensively consolidated ID1 and ID2 in the Mie and Gifu datasets. The left graph (a) reports the fraction histogram of the number of ID1 values merged into a single vPID value, and the right graph (b) reports the same histogram for ID2. These graphs indicate that 17.1% and 15.9% of the patients had multiple ID1 values in the Mie and Gifu datasets; vPID allowed to trace the claims of those patients thoroughly, even though ID1 could not do that. Similarly, 46.8% and 39.3% of the patients had multiple ID2 values; vPID enables tracing those patients’ claims.

### 3.3 Identifier cardinalities in the national-level dataset

**Fig. 3:**
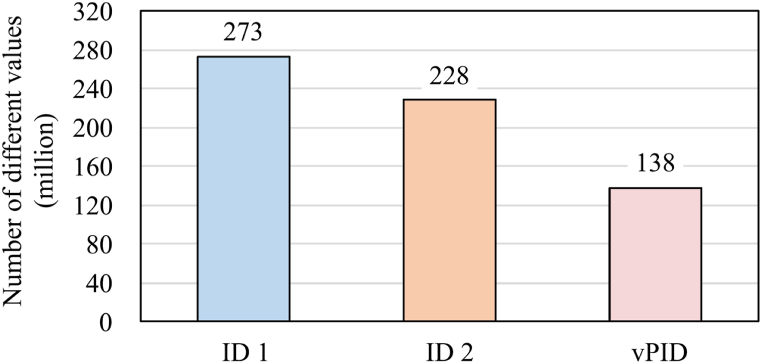
Numbers of different values (cardinalities) of different identifiers in the NDB dataset.

Next, we applied the vPID algorithm to the NDB dataset to clarify how the existing identifiers (ID1 and ID2) were merged in the NDB dataset. Fig. 3 reports the cardinalities (i.e., number of different values) for ID1, ID2, and vPID in the NDB dataset, implying that vPID helped to collect all the claims for the majority of the covered citizens. The NDB dataset held 273 million and 228 million different values for ID1 and ID2, respectively, whereas vPID condensed them up to 138 million values. This number was significantly closer to the actual population of Japan (127 million, 2014).

### 3.4 Case study: a national-level longitudinal analysis of health state transition

Finally, we present another case study of longitudinal analysis, wherein we applied the vPID algorithm to the NDB dataset. Table 3 illustrates a longitudinal analysis regarding how the health status of the Japanese population changed over time. First, we retrieved patients to be recognized as “Grade 0” (having no lifestyle diseases) from the FY2009 (the fiscal year 2009; April 2009 through March 2010) subset of the NDB dataset. As a result, 101 million “Grade 0” patients were identified in the FY2009 subset. Second, we traced their claims forward year by year and grouped their health states into five grades: Grade 0, Grade 1 (being diagnosed with any lifestyle diseases), Grade 2 (taking medication for any lifestyle diseases), Grade 3 (having complications, such as ischemic heart disease or stroke), and Grade 4 (having a low quality of life with dialysis). The detailed definition of each grade is presented in Supplementary Tables S1–4. Table 3 indicates the temporal deteriorating process of the health status of the “Grade 0” patients of FY2009.

**Table 3:** Analysis of health state transition of patients having no lifestyle disease in FY2009 (the fiscal year 2009; April 2009 through March 2010).

		FY2009	FY2010	FY2011	FY2012	FY2013	FY2014
**Number of patients (thousand)**	Grade 0	101,118	91,161	88,592	86,720	84,945	83,361
	Grade 1	–	2862	3306	3574	3820	4093
	Grade 2	–	2533	3547	4293	4952	5492
	Grade 3	–	4546	5651	6504	7371	8135
	Grade 4	–	17	22	26	31	37

Lifestyle disease: diabetes, hyperlipidemia, or hypertension.

Grade 0: having no lifestyle disease.

Grade 1: being diagnosed with any lifestyle disease.

Grade 2: taking medication for any lifestyle disease.

Grade 3: having complications, such as ischemic heart disease or stroke.

Grade 4: having a low quality of life with dialysis.

FY: Fiscal year.

## 4 Discussion

Kubo et al. presented yet another method for merging ID1 and ID2 on NDB, and demonstrated the accuracy improvement by presenting the predicted Japanese population [19]. Our present study has formally defined two quantitative accuracy indicators, and compared vPID with the ground truth. To our knowledge, this is the first report in the literature.

Researchers actively studied how to merge patient records in healthcare databases [[31], [32], [33], [34], [35], [36], [37], [38], [39], [40], [41], [42], [43], [44], [45], [46], [47], [48], [49], [50], [51], [52], [53], [54], [55], [56], [57], [58], [59], [60], [61], [62], [63]]. These studies can be roughly grouped into three approaches: a deterministic approach [[31], [32], [33], [34], [35], [36], [37], [38], [39], [40], [41], [42], [43], [44], [45], [46]], a probabilistic approach [[47], [48], [49], [50], [51], [52], [53], [54], [55], [56], [57], [58]], and a hybrid approach [[59], [60], [61], [62], [63]]. Our method can be categorized into the deterministic approach, but it can be extended by incorporating other approaches.

Even though vPID is useful for a wide spectrum of analyses in practice, we need to consider possible limitations. First, a substantial identification error was observed only on the Gifu dataset. The specific property of this dataset seemed to pollute vPID. The Gifu dataset contained some erroneous claims, which had identical incorrect values (e.g., blank name and zero birthdate). Such claims produced the same incorrect ID1/ID2 value for different patients. Hence, vPID happened to merge such different patients by mistake. We are attempting to remove such erroneous claims in the preprocessing process by utilizing heuristic rules and machine learning techniques. In addition, we are also investigating the remarkable inter-prefecture discrepancy (e.g., 0.602, Mie and 0.839, Gifu of ID2 in traceability) observed in the verification test.

Second, the present study verified the quality of vPID with a dataset of four years. The observed result might have been biased by this dataset period limitation. We need further investigation with a dataset of a much longer time period, since longitudinal healthcare studies need to be robust over a long time period.

Third, vPID cannot merge ID1 or ID2 of a citizen for whom no healthcare insurance claims are recorded because vPID is obtained from a claim dataset. In our experience [[13], [14], [15], [16], [17], [18]], many healthcare analysts are primarily interested in the reality of healthcare service provision to patients. This limitation likely has a negligible impact on such studies that do not focus on the citizens being unserviced of healthcare.

Fourth, vPID cannot merge a patient's claims if its ID1 and ID2 change simultaneously. For example, when a couple gets married, either of the couple may change their workplaces and change their family names on the same day, thus causing the simultaneous change of ID1 and ID2. The presented verification test has clarified that vPID offers significantly high traceability scores (0.994–0.997) overall. Only 4875 (0.582%) and 3221 (0.319%) insured citizens in Mie and Gifu, respectively, were given multiple vPID values due to the simultaneous change of ID1 and ID2 values. This indicates that this limitation is also negligible for studies that do not focus on a patient group who experiences such simultaneous life events (e.g., marriage and job change at the same time).

Finally, vPID inherits an identifiability issue that ID1 originally holds. Some Japanese public healthcare insurers assign a unique insured identifier for each family, instead of each family member; the family members may have the same insured identifier. Even in such a case, different members usually have a different combination of genders and birthdates; a unique ID1 is usually assigned for each member. A typical exceptional case is in same-sex twin children, who are highly likely to stay in the same family and have the same birthdate. The same ID1 value can be assigned for both twin children; ID1 cannot distinguish such twin children. Being developed on top of ID1, vPID still holds this negative nature. Our investigation confirmed that 885 (0.0945%) and 954 (0.0939%) vPID values were faultily shared by multiple insured citizens in the Mie and Gifu datasets, respectively. This limitation can be considered to be negligible for many studies [55,[64], [65], [66]], unless they focus on twins. On the other hand, twins attract the attention of specific healthcare studies. The use of recent machine learning techniques might be helpful. Our latest attempt is reported in Supplementary Section S4.

This paper has studied the identifier consolidation in homogeneous data space. Extending the study to heterogeneous space is unexplored yet. Combining healthcare insurance claims and electronic health records potentially helps capture new knowledge [36,67]. Both the datasets are not necessarily anonymized in a perfectly consistent fashion. We need a tangible method to merge identifiers among the different datasets.

Anonymization is an essential technical method to allow the society to effectively utilize healthcare data for improving its healthcare system and safely protect the privacy simultaneously. In most data delivery processes, data is firstly anonymized by the original owners when it is delivered to third-party data analysts; legal regulations and leakage risks are the primary factors in practice for the data owners to determine the anonymization design. Hence, the anonymized data may hold quality issues for the data analysts, since their quality preferences or requirements are not deeply or never considered in the anonymization process. This problem potentially happens in many healthcare data delivery systems; the identifier issue of the Japanese NDB addressed in this paper is one typical case. From a technical aspect, taking two concurrent approaches seems reasonable. The first approach is to redesign the data delivery process from scratch or renovate the existing data delivery process in order to resolve the recognized data quality issues. However, this solution often needs considerable time when the data delivery process is large-scale and distributed. For example, if we try to change the anonymization rule employed in NDB, we need to achieve a new consensus between its stakeholders and implement the new rule consistently throughout the data delivery process. This approach is actually orthodox, but merely relying on it is not a good idea in practice because significant opportunities are lost for data analysts. Thus, we need the second approach, which is to explore the way to exploit the existing data delivery process and the organized database; they have some data quality issues in part, but they have the potential to offer new findings. Our attempt presented in this paper takes the second approach; vPID has been developed to compensate the information (i.e., patient traceability) lost by the anonymization process in NDB.

Data management of national healthcare systems is actively studied because it has been deemed to be a key factor towards better healthcare systems. Existing healthcare systems may not be necessarily compatible or with the digital technology; the poor patient traceability addressed in this paper is one example. The society needs to continuously reform the healthcare regulations to be able to exploit the technology innovation. For example, Japan's public healthcare insurance system is currently composed of multiple distributed subsystems; the patient identifier is not unitary. Assuming that a unitary patient identifier was employed in Japan, it would simplify the patient identification and tracing work, thus improving the quality and productivity of Japanese healthcare analysis. Similarly, the Japanese government is allowed to access limited parts out of the data held by public insurers. If a strong responsibility of data preserving and sharing were thoroughly enforced in the healthcare system, it would facilitate the data acquisition work, thus extending the potential of healthcare analysis. In spite of their benefits, such new regulations often impose the additional technical expense and reduce the social flexibility. Social consensus is essential; only the visible benefits that data analysis enabled by those regulations provides to the people (e.g., the improvement of healthcare systems) can justify the technical and social cost. Leaders in the fields of healthcare and computer science need to keep their efforts to present specific visible benefits to the people in parallel to purely technical benefits.

## 5 Conclusion

This study has proposed the virtual patient identifier (vPID), a new composite identifier on top of the existing anonymized identifiers (ID1 and ID2) in the Japanese national-level healthcare insurance claims database (NDB). vPID intensively merges ID1 and ID2 that co-occur in identical claims in the database. The comparative verification with the Mie and Gifu datasets has clarified that vPID offers significantly higher traceability scores (0.994–0.997) with comparably high (0.996, Mie) and lower (0.979, Gifu) identifiability scores. vPID provides an opportunity for longitudinal analyses that used to be practically impossible on NDB. Further exploration is necessary, particularly for mitigating identification errors observed in the Gifu dataset.

## Author contribution statement

Jumpei Sato, Kazuo Goda: Conceived and designed the experiments; Performed the experiments; Analyzed and interpreted the data; Contributed reagents, materials, analysis tools or data; Wrote the paper. Hiroyuki Yamada, Masaru Kitsuregawa: Contributed reagents, materials, analysis tools or data. Naohiro Mitsutake: Contributed reagents, materials, analysis tools or data; Wrote the paper.

## Data availability statement

The authors do not have permission to share data.

## Declaration of competing interest

The authors declare that they have no known competing financial interests or personal relationships that could have appeared to influence the work reported in this paper.

## Appendix A Supplementary data

Supplementary_File_k_1 Supplementary_File_k_1
