# Supplementary material for: Virtual patient identifier (vPID): Improving patient traceability using anonymized identifiers in Japanese healthcare insurance claims database
Source: Heliyon. 2023 May 12;9(5):e16209. doi: 10.1016/j.heliyon.2023.e16209 (PMC10205637; doi:10.1016/j.heliyon.2023.e16209)
Supplement: Supplementary_File_k_1 [file mmc1.pdf]

SUPPLEMENTARY MATERIALS

S1. Definition of a generation algorithm of virtual patient identifiers (vPIDs)

Supplementary Figure S1 formally presents an algorithm that we have developed to generate vPID for a given claims database. Let C be a set of healthcare insurance claims, where a claim c $(c \in C)$ is provided with two different anonymized patient identifiers, specifically ID1 c_{ID1} and ID2 c_{ID2}. Let (V, E) be a graph, where V is a vertex set, and E is an edge set. V and E are initially empty. First, the algorithm (lines 3–5) retrieves all possible combinations of identifiers (c_{ID1}, c_{ID2}) from the database and stores the combinations as vertexes into V. Second, the algorithm (lines 6–12) identifies all possible edges between two vertexes having the same values for either of c_{ID1} and c_{ID2} and stores the edges into E. Finally, the algorithm (lines 13–16) enumerates all disconnected components in the graph (V, E) and assigns a unique value (vPID) for each disconnected component.

vPID can be utilized as a new identifier for a unique patient, and the graph (V, E) can be utilized as an index for searching healthcare insurance claims; for a given vPID value, one can enumerate all the combinations (c_{ID1}, c_{ID2}) in an associated disconnected component, thus retrieving all the associated claims from the database.

Algorithm 1 Algorithm for generating vPIDs for a claim set C

1: $V \leftarrow \emptyset$ ▷ V is a vertex set
2: $E \leftarrow \emptyset$ ▷ E is a edge set
3: **for all** $c \in C$ **do**
4:     Put a vertex $(c_{ID1,ID2}) \rightarrow V$
5: **end for**
6: **for all** $(i_1, i_2) \in V$ **do**
7:     **for all** $(j_1, j_2) \in V$ **do**
8:         **if** $(i_1 = j_1 \wedge i_2 \neq j_2) \vee (i_1 \neq j_1 \wedge i_2 = j_2)$ **then**
9:             Put an edge $((i_1, i_2), (j_1, j_2)) \rightarrow E$
10:         **end if**
11:     **end for**
12: **end for**
13: **for all** $g \in (V, E)$ **do** ▷ Enumerating connected components g from a graph (V, E)
14:     Assign a unique value p to g ▷ p is a vPID for g
15:     Put $(p, g) \rightarrow G$
16: **end for**
17: **return** G

Supplementary Figure S1. Algorithm for generating virtual patient identifiers (vPIDs) from a given claims database.

S2. Formal definition of validation indicators

Suppose a given identifier system S. Let E be a set of enrollee history records, where a e ($e \in E$) is provided with an anonymized patient identifier $e_{i(S)}$ and a unique citizen number e_n. Let I(S) be a set of all possible anonymized patient identifiers, $I(S) = \{e_{i(S)} | \forall e \in E\}$, and N be a set of all possible anonymized citizen numbers, $N = \{e_n | \forall e \in E\}$.

The following two indicators quantitatively evaluate the accuracy of the identifier system S.
The identifiability score:

$$\frac{\left|\left\{i \middle| \forall i \in I(S) \wedge \left|\left\{e_n \middle| \forall e \in E \wedge e_{i(S)} = i\right\}\right| = 1\right\}\right|}{|I(S)|} \tag{1}$$

indicates the capability of distinguishing every patient's claims from the others patients' claims, and the traceability score:

$$\frac{\left|\left\{n \middle| \forall n \in N \wedge \left|\left\{e_{i(S)} \middle| \forall e \in E \wedge e_n = n\right\}\right| = 1\right\}\right|}{|N|} \tag{2}$$

indicates the capability of collecting all the claims of every patient. Note that the notation $|A|$ denotes a norm of a set A. Obviously, both scores take values between 0 and 1; greater values indicate higher accuracy.

S3. Definition of patient grades for the case study of a nation-level longitudinal analysis of health state transition

Supplementary Tables S1–4 present the detail definition of each grade in the case study presented in Section 3.4.

Supplementary Table S1. Definition of different patient grades used in the second case study.

Grade	Definition
Grade 0	Patients who were not recognized to be any other grades.
Grade 1	Patients who were diagnosed with any of the lifestyle diseases (diabetes, hyperlipidemia and hypertension) listed in Supplementary Table S2, but not recognized to be Grade 2, Grade 3 or Grade 4 during a concerned time period.
Grade 2	Patients who were prescribed with any of the drugs listed in Supplementary Table S3, but not recognized to be Grade 3 or Grade 4 during a concerned time period.
Grade 3	Patients who were diagnosed with any of complicating diseases (ischemic heart disease, stroke, diabetic retinopathy, and chronic kidney disease) listed in Supplementary Table S2, but not recognized to be Grade 4 during a concerned time period.
Grade 4	Patients who received any of dialysis treatments listed in Supplementary Table S4 during a concerned time period.

Supplementary Table S2. Diagnosis codes for each of the seven diseases (diabetes, hyperlipidemia, hypertension, ischemic heart disease, stroke, diabetic retinopathy, and chronic kidney disease) used in the case study and the ICD10 codes to which the diagnosis codes belong.

Disease name	ICD10 code	Diagnosis code
Diabetes	E10, E100–E107, E109, E11, E110–E117, E119, E12, E13, E130–E137, E139, E14, E140–E146, E149	2500001, 2500013, 2500014, 2500015, 2500024, 2500027, 2500037, 2500041, 2501002, 2501003, 2501005, 2502004, 2502006, 2503005, 2503007, 2504004, 2504005, 2504006, 2504010, 2504012, 2504013, 2505011, 2505018, 2505021, 2506006, 2506011, 2507025, 2507028, 2507029, 2509003, 2509004, 8830028, 8830030, 8830031, 8830032, 8830033, 8830040, 8830041, 8830042, 8830043, 8830044, 8830045, 8830405, 8830756, 8832747, 8835244, 8836564, 8838063, 8838064, 8838065, 8838066, 8838067, 8838068, 8838069, 8838070, 8838071, 8838072, 8838073, 8838074, 8838075, 8838076, 8838077, 8838078, 8838079, 8838080, 8840710, 8841679, 8841680, 8841681, 8841682, 8841683, 8841684, 8841685, 8841686, 8841687, 8841688, 8841689, 8841690, 8841691, 8841692, 8841693, 8841694, 8841695, 8841696, 8841697, 8841698, 8843105, 8843106, 8843120, 8843121, 8843122, 8843123, 8843124, 8843125, 8843126, 8843127, 8843128, 8843375, 8843376, 8843377, 8843378, 8843379, 8843380, 8843381, 8843382, 8843383, 8843388, 8843389, 8843390, 8843391, 8843392, 8843393, 8843394, 8843395, 8843396, 8843439, 8843448, 8843449, 8843450, 8843451, 8843452, 8843453, 8843454, 8843455, 8843456, 8843619, 8843620, 8843621, 8843622, 8843623, 8843624, 8843625, 8843626, 8843627, 8843982, 8843983, 8843984, 8843985, 8843986, 8843987, 8843988, 8843989, 8843990, 8843991, 8843992, 8843993, 8843994, 8843995, 8843996, 8843997, 8844022, 8844023, 8844024, 8844025, 8844026, 8844027, 8844028, 8844029, 8844030, 8844031,

Disease name	ICD10 code	Diagnosis code
		8844045, 8844089, 8844346, 8844347, 8844536, 8844537, 8844626, 8844627, 8844628, 8844629, 8844652, 8844653, 8845043, 8845044, 8845045, 8845046, 8845047, 8845048, 8845049, 8845050, 8845051, 8845052, 8845053, 8845054, 8845055, 8845056, 8845057, 8845058, 8845059, 8845060, 8845061, 8845062, 8845063, 8845064, 8845065, 8845066, 8845067, 8845068, 8845069, 8845070, 8845071, 8845072, 8845073, 8845074, 8845075, 8845076, 8845077, 8845078, 8845079, 8845080, 8845081, 8845082, 8845083, 8845084, 8845085, 8845086, 8845087, 8845088, 8845089, 8845090, 8845091, 8845092, 8845093, 8845094, 8845095, 8845096, 8845097, 8845098, 8845099, 8845100, 8845128, 8845198, 8845842
Hyperlipidemia	E780–E785	2720001, 2720004, 2721002, 2723001, 2724007, 2724012, 2724023, 2724031, 2724036, 2724037, 8831269, 8831270, 8831271, 8831272, 8831273, 8831274, 8831275, 8831286, 8833120, 8833435, 8833722, 8833881, 8840108, 8844446, 8845523, 8845524
Hypertension	I10, I110, I119–I120, I129, I139, I150–I152, I159	4019016, 4019017, 4029010, 4039001, 4039005, 4039006, 4039025, 4039026, 4039028, 4039033, 4039036, 5879003, 8830212, 8832479, 8833421, 8833422, 8833425, 8833426, 8833427, 8835586, 8835605, 8835614, 8838336, 8838398, 8839689, 8840107, 8842089, 8842094, 8842178, 8842488, 8842500
Ischemic heart disease	I200–201, I208–I214, I219–I221, I228–I236, I240–I241, I248–I249, I251–I256, I258–I259	4100008, 4109038, 4109056, 4110003, 4119015, 4119019, 4139004, 4139007, 4139023, 4139026, 4139028, 4140008, 4140013, 4140014, 4140020, 4141001, 4141003, 4141004, 4148001, 4149007, 8830155, 8830403, 8831448, 8831572, 8831573, 8831574, 8831575, 8831576, 8831577, 8831578, 8831579, 8832297, 8832298, 8832310, 8832316,

Disease name	ICD10 code	Diagnosis code
		8832326, 8832330, 8832338, 8832347, 8832348, 8832356, 8832357, 8832376, 8832377, 8832378, 8832379, 8832380, 8832381, 8832382, 8832383, 8832384, 8832385, 8832387, 8832389, 8832400, 8832401, 8832402, 8832403, 8832404, 8832409, 8832508, 8832678, 8833095, 8833544, 8833682, 8833961, 8833962, 8833963, 8833965, 8833966, 8833970, 8834877, 8834919, 8836577, 8836578, 8836815, 8836816, 8837558, 8837801, 8837804, 8837807, 8837809, 8837810, 8838289, 8840493, 8841211, 8841212, 8842211, 8842459, 8842460, 8842490, 8842693, 8842760, 8842762, 8843284, 8844146, 8844586, 8845119, 8845239, 8846370, 8846372, 8846988, 8847004, 8847005, 8847013, 8847031, 8847032, 8847036, 8847038, 8847039, 8847040, 8847041, 8847057, 8847058, 8847059, 8847060, 8847530, 8847838
Stroke	I600–I611, I613–I616, I618–I621, I629–I636, I638–I639, I64, I671–I679, I690–I691, I693–I694,	3259002, 3259015, 3448002, 3448022, 3448028, 3458023, 3489005, 3489029, 3489032, 3489035, 4309001, 4309005, 4310038, 4319003, 4319006, 4319009, 4319013, 4319018, 4319020, 4319027, 4319030, 4319032, 4320007, 4321006, 4321008, 4330012, 4341002, 4341005, 4341010, 4341018, 4341026, 4341027, 4341044, 4341045, 4341049, 4341050, 4341052, 4341053, 4341056, 4349005, 4369009, 4369014, 4369016, 4370003, 4370011, 4371003, 4372001, 4372002, 4372003, 4373003, 4373005, 4373007, 4373010, 4373012, 4373014, 4373015, 4373016, 4373020, 4373023, 4373028, 4374003, 4375001, 4375003, 4375004, 4378007, 4378020, 4379006, 4379010, 4379014, 4379015, 4389001, 4389014, 4389017, 4389024, 4389027, 4428003, 8830739, 8830740, 8832970, 8833433, 8833591, 8833690, 8834808, 8834968, 8835412, 8835486, 8835797, 8836339, 8836504, 8836998,

Disease name	ICD10 code	Diagnosis code
		8837619, 8837825, 8838299, 8838688, 8838690, 8838691, 8838692, 8838694, 8838703, 8838704, 8838708, 8838709, 8838721, 8838722, 8838740, 8838749, 8838751, 8838753, 8838770, 8839201, 8839202, 8839253, 8839257, 8841358, 8842228, 8842229, 8842255, 8842272, 8842527, 8842858, 8842952, 8843057, 8843499, 8843500, 8843753, 8844427, 8844432, 8844485, 8844502, 8844561, 8845147, 8846034, 8846321, 8846351, 8846352, 8846357, 8846358, 8846373, 8846374, 8846384, 8846385, 8846397, 8846410, 8846412, 8846413, 8846414, 8846415, 8846416, 8846417, 8846419, 8846420, 8846424, 8846425, 8846426, 8846427, 8846428, 8846429, 8846430, 8846431, 8846436, 8846437, 8846438, 8846439, 8846440, 8846450, 8846451, 8846593, 8846750, 8847449, 8847468, 8847469, 8847505, 8847506, 8847527, 8847531, 8847536, 8847541, 8847545, 8847569, 8847680, 8847851, 8847895, 8847896, 8847897, 8848096, 9009002
Diabetic retinopathy	E103, E113, E143	2504005, 2504010, 2504013, 8830033, 8830045, 8836564, 8844536, 8844537, 8845064
Chronic kidney disease	E102, E112, E132, E142, I129, I139, N028, N030, N032–N034, N037, N039, N040, N042, N044, N046, N049, N050–N059, N079, N180, N188–N189, N19, N26, N289, R944	2503005, 2503007, 4039001, 4039005, 4039006, 4039015, 4039018, 4039025, 4039028, 4039033, 4039038, 5819004, 5829003, 5829008, 5831001, 5839007, 5839010, 5839017, 5839033, 5859001, 5859002, 5860004, 5869015, 5869016, 5879003, 5939017, 7533005, 7944001, 8830031, 8830042, 8831660, 8832747, 8832844, 8832919, 8833425, 8834241, 8834799, 8835584, 8835590, 8835642, 8835738, 8836335, 8836667, 8836820, 8837974, 8838071, 8838367, 8838401, 8838554, 8838555, 8838994, 8839430, 8839471, 8839535, 8839551, 8840228, 8840229, 8840230, 8840231, 8840232, 8840391, 8840392, 8840393, 8840394, 8840538,

Disease name	ICD10 code	Diagnosis code
		8841385, 8841681, 8841691, 8843124, 8843379, 8843392, 8843452, 8843623, 8843983, 8843984, 8843985, 8843986, 8843987, 8843988, 8843989, 8843991, 8843992, 8843993, 8843994, 8843995, 8843996, 8843997, 8844028, 8844106, 8845058, 8845059, 8845087, 8845088, 8847544, 8847577, 8847578, 8847579, 8847580, 8847581, 8847582, 8847583, 8848070, 8848361, 8848410

Supplementary Table S3. Drug codes for each of the three diseases (diabetes, hyperlipidemia, and hypertension) used in the case study and the ATC codes to which the drug codes belong.

Indication	ATC code	Drug code
Diabetes	A10H0, A10J1, A10K1, A10K2, A10K3, A10L0, A10M1, A10M9, A10N1, A10N9, A10P0, A10X9, A10C1–A10C3, A10C5, A10S0	610406390, 610406391, 610407055, 610412056, 610432026, 610432027, 610432032, 610432033, 610432040, 610432041, 610433079, 610441043, 610443002, 610443003, 610444147, 610463145, 613960002, 613960003, 613960015, 613960017, 613960020, 613960027, 613960032, 613960036, 613960038, 613960039, 613960041, 613960057, 613960060, 613960067, 613960072, 613960074, 613960077, 613960078, 613960079, 613960081, 613960082, 620000048, 620000203, 620000205, 620000265, 620000266, 620000267, 620000268, 620000269, 620000270, 620000271, 620000442, 620000443, 620000447, 620000448, 620001907, 620001908, 620002029, 620002030, 620002031, 620002032, 620002120, 620002121, 620002439, 620002440, 620002441, 620002442, 620002443, 620002444, 620002445, 620002661, 620002662, 620002663, 620002664, 620002665, 620002666, 620002667, 620002668, 620002711, 620002712, 620002714, 620002715, 620002716, 620002717, 620002725, 620002730, 620002731, 620002810, 620002812, 620002813, 620002815, 620002816, 620002824, 620002825, 620002826, 620002827, 620002828, 620002829, 620002835, 620002836, 620002837, 620002838, 620002839, 620002840, 620002841, 620002842, 620002843, 620002844, 620002845, 620002846, 620002847, 620002848, 620002859, 620002862, 620003127, 620003128, 620003129, 620003143, 620003159, 620003160, 620003277, 620003452, 620003568, 620003604, 620003661, 620003947, 620003948, 620004029, 620004045, 620004069, 620004070, 620004071, 620004072, 620004073, 620004074, 620004289,

Indication	ATC code	Drug code
		620004474, 620004480, 620004482, 620004502, 620004580, 620004781, 620004842, 620005359, 620005360, 620005557, 620005558, 620005559, 620005560, 620005561, 620005562, 620005563, 620005564, 620005565, 620005566, 620005570, 620005885, 620005900, 620005901, 620005979, 620006030, 620006590, 620006682, 620006683, 620006872, 620006890, 620006891, 620007459, 620007460, 620007461, 620007462, 620007536, 620008071, 620008072, 620008073, 620008074, 620008075, 620008076, 620008726, 620008727, 620008728, 620008729, 620008893, 620008894, 620008895, 620008896, 620008897, 620008898, 620008899, 620008907, 620008908, 620008909, 620008910, 620008911, 620008912, 620008913, 620008914, 620008915, 620008916, 620008932, 620008933, 620008934, 620008935, 620008936, 620008942, 620008943, 620008944, 620008945, 620008952, 620008953, 620009133, 620009209, 620009286, 620009287, 620009288, 620009289, 620009290, 620009291, 620009292, 620009293, 620009294, 620009295, 620009296, 620009297, 620871601, 620872001, 620872002, 620872003, 620872004, 620872009, 620873202, 620873301, 620873402, 620873702, 621665301, 621665401, 621673401, 621673501, 621673601, 621676001, 621678801, 621678901, 621679001, 621682301, 621682402, 621683201, 621683401, 621683501, 621689001, 621689101, 621689303, 621689403, 621690203, 621690303, 621690402, 621690502, 621690901, 621691001, 621691201, 621691601, 621748301, 621911101, 621911201, 621911301, 621926901, 621927001, 621937101, 621937201, 621942101, 621942102, 621942201, 621942202, 621943301, 621943401, 621950901, 621951001,

Indication	ATC code	Drug code
		621951101, 621953301, 621953401, 621958701, 621958801, 621970601, 621970701, 621970801, 621973201, 621973301, 621974701, 621974801, 621980701, 621982701, 621986001, 621986101, 621986201, 621986301, 621986401, 621990901, 621991001, 621997001, 621998701, 621998801, 621998901, 621999001, 621999301, 621999401, 621999701, 621999801, 622000601, 622000701, 622001701, 622001801, 622004701, 622004801, 622005501, 622005601, 622008501, 622008502, 622008601, 622008602, 622008701, 622008801, 622009801, 622009802, 622009901, 622010001, 622011401, 622011501, 622011601, 622011701, 622013401, 622013501, 622013601, 622016001, 622016101, 622017301, 622017401, 622017501, 622017901, 622018001, 622018801, 622018802, 622020901, 622020903, 622021001, 622021003, 622021801, 622021901, 622022001, 622022101, 622023501, 622023601, 622025201, 622025301, 622025801, 622025901, 622026501, 622026601, 622029901, 622030001, 622031401, 622031501, 622033001, 622033101, 622033201, 622033701, 622033801, 622035701, 622035801, 622036001, 622036002, 622037901, 622038001, 622038301, 622038401, 622038801, 622040901, 622041001, 622041202, 622041302, 622041402, 622041502, 622042901, 622043001, 622045201, 622045301, 622045401, 622046801, 622046901, 622047701, 622047801, 622048401, 622048501, 622049901, 622050001, 622053101, 622053201, 622053601, 622053801, 622055801, 622055901, 622056001, 622056101, 622058801, 622058901, 622059001, 622059002, 622059101, 622059102, 622059201, 622059301, 622061001, 622061401, 622061501, 622061601, 622061701, 622062301, 622062302,

Indication	ATC code	Drug code
		622062401, 622062402, 622063001, 622063101, 622063201, 622063301, 622065101, 622065201, 622065301, 622065401, 622066201, 622066301, 622070801, 622071701, 622071801, 622071901, 622072001, 622075601, 622078301, 622078401, 622079101, 622079201, 622081801, 622081901, 622086001, 622086101, 622088001, 622088301, 622088401, 622090001, 622090101, 622093501, 622103201, 622114401, 622114501, 622114601, 622114701, 622114801, 622118501, 622119301, 622119401, 622122201, 622122301, 622127301, 622127401, 622127501, 622128101, 622137701, 622141301, 622141302, 622143401, 622143402, 622144001, 622144701, 622147301, 622147401, 622147501, 622147601, 622155701, 622155801, 622155901, 622156001, 622156901, 622159301, 622159401, 622163301, 622163401, 622164401, 622166801, 622166901, 622167201, 622167301, 622169101, 622169102, 622169301, 622171301, 622172101, 622172201, 622175401, 622175501, 622175701, 622176301, 622177501, 622178601, 622178701, 622182601, 622186201, 622187301, 622190001, 622193301, 622194901, 622196601, 622196701, 622198001, 622198901, 622199001, 622201701, 622202201, 622202801, 622205101, 622205501, 622208901, 622211501, 622217701, 622219701, 622221001, 622222001, 622229001, 622230001, 622230101, 622242001, 622242501, 622245601, 622245701, 622246801, 622252501, 622252701, 622254701, 622267001, 622271101, 622271201, 622271301, 622277501, 622288401, 622306601, 622306701, 622313200, 622320800, 622335701, 622335801, 622336801, 622338501, 622338601, 622340101, 622341901, 622342001, 622360601, 622401201, 622401301, 622406001,

Indication	ATC code	Drug code
		622410901, 622411001, 622412701, 622415401, 622415501, 622417101, 622417201, 622421101, 622421201, 622421901, 622422001, 622424401, 622426601, 622426701, 622427201, 622427301, 622432601, 622432701, 622436301, 622438401, 622438501, 622440701, 622442201, 640406239, 640407220, 640407221, 640407222, 640412080, 640412081, 640412082, 640412083, 640412084, 640412085, 640422067, 640422068, 640422069, 640422074, 640451027, 640451028, 640451029, 640451038, 640451040, 640451041, 640453021, 640453022, 640453023, 642490059, 642490061, 642490107, 642490121, 642490123
Hyperlipidemia	C10A1–C10A3, C10A9, C10B0	610407028, 610421343, 610421344, 610422004, 610422023, 610422031, 610422053, 610422054, 610422055, 610422056, 610422057, 610422059, 610422098, 610422123, 610422206, 610422221, 610422258, 610422259, 610422261, 610422262, 610422263, 610422264, 610422265, 610422276, 610422286, 610422290, 610422292, 610432003, 610433008, 610433091, 610433110, 610433125, 610433126, 610433131, 610443013, 610443014, 610454084, 610454085, 610462007, 610462015, 610462016, 610463087, 610470012, 610470013, 610470014, 612180004, 612180011, 612180028, 612180106, 612180124, 612180140, 612180141, 612180183, 612180186, 612180194, 612180198, 612180206, 612180213, 612180231, 612180232, 612180263, 612180264, 612180265, 612180271, 612180272, 612180280, 612180282, 612180283, 612180288, 612180290, 612180291, 612190001, 612190002, 612190067, 612190069, 612190079, 612190172, 612190187, 612190258, 613390006, 620000013, 620000014, 620000038, 620000039, 620000051, 620000052, 620000053, 620000054,

Indication	ATC code	Drug code
		620000055, 620000070, 620000071, 620000103, 620000104, 620000105, 620000106, 620000107, 620000108, 620000109, 620000110, 620000111, 620000112, 620000113, 620000114, 620000115, 620000116, 620000140, 620000141, 620000152, 620000153, 620000156, 620000157, 620000158, 620000159, 620000160, 620000163, 620000164, 620000169, 620000171, 620000174, 620000175, 620000176, 620000177, 620000178, 620000179, 620000422, 620000423, 620001883, 620001924, 620002049, 620002050, 620002052, 620002108, 620002109, 620002117, 620002118, 620002123, 620002162, 620002163, 620002164, 620002165, 620002166, 620002423, 620002424, 620002433, 620002434, 620002477, 620002478, 620002508, 620002540, 620002541, 620002736, 620002798, 620002799, 620002800, 620002801, 620002802, 620002879, 620002880, 620003668, 620003669, 620004037, 620004038, 620004459, 620004519, 620004607, 620004608, 620004609, 620004868, 620004942, 620004985, 620005032, 620005513, 620005785, 620005931, 620005932, 620005938, 620005965, 620006039, 620006102, 620006108, 620006115, 620006601, 620006681, 620006870, 620006904, 620007887, 620007889, 620007890, 620007891, 620007892, 620007893, 620007894, 620007895, 620007896, 620007897, 620007898, 620007993, 620007994, 620007995, 620008053, 620008054, 620008055, 620008056, 620008112, 620008113, 620008508, 620008610, 620008631, 620008669, 620008679, 620008710, 620008784, 620009208, 620009218, 620009322, 620009323, 620009425, 620009426, 620338305, 620338317, 620339201, 620339401, 620340201, 620340602, 620340603, 620340901, 620341301, 620344306,

Indication	ATC code	Drug code
		620346001, 620346008, 620346023, 620346029, 620346101, 620353201, 620353202, 620353601, 620353804, 620815601, 620815903, 620816201, 620816501, 620816805, 620817504, 620817702, 621254601, 621523101, 621523201, 621524102, 621524402, 621525701, 621525801, 621528602, 621528702, 621528801, 621528901, 621529001, 621529101, 621531001, 621531101, 621531703, 621532401, 621532501, 621532902, 621533002, 621533101, 621533201, 621533601, 621533801, 621533901, 621534101, 621534301, 621635202, 621639001, 621639101, 621639701, 621639801, 621643301, 621643401, 621643501, 621643601, 621675101, 621694001, 621934801, 621934901, 621935001, 621948701, 621955001, 621959901, 621960001, 621960101, 621964101, 621964201, 621964301, 621964401, 621964501, 621964601, 621981401, 621981403, 622015101, 622015201, 622015301, 622039501, 622039601, 622052801, 622055602, 622071601, 622075801, 622076401, 622076501, 622090701, 622090801, 622096101, 622096102, 622096801, 622096901, 622098401, 622098501, 622099101, 622107601, 622107701, 622110401, 622110501, 622116802, 622126901, 622127001, 622136401, 622139600, 622143801, 622143901, 622152001, 622152101, 622161801, 622161901, 622165601, 622165701, 622167601, 622167701, 622169901, 622170101, 622170201, 622180601, 622180701, 622186601, 622186701, 622187601, 622187701, 622198801, 622204801, 622204901, 622217101, 622217201, 622239201, 622239301, 622241301, 622241401, 622244801, 622244901, 622252001, 622252101, 622268001, 622268101, 622269101, 622269201, 622270001, 622270101, 622271801, 622271901, 622273101,

Indication	ATC code	Drug code
		622273201, 622273301, 622274901, 622275001, 622276301, 622276401, 622280201, 622280301, 622280401, 622280501, 622280601, 622280701, 622280801, 622282201, 622283701, 622283801, 622285001, 622285101, 622286201, 622286301, 622286401, 622287601, 622289501, 622289601, 622291801, 622291901, 622292001, 622292301, 622292401, 622292501, 622293301, 622293401, 622294301, 622294401, 622296001, 622296101, 622296201, 622297101, 622297201, 622298001, 622298101, 622299001, 622299101, 622302501, 622302801, 622302901, 622304701, 622304801, 622304901, 622315400, 622347401, 622359101, 622360201, 622365801, 622372401, 622387601, 622419701, 622419801, 622421601, 622421701, 622434301, 622434401
Hypertension	C02A1–C02A3, C02C0, C02D0, C03A1–C03A3, C03A7, C03A9, C07A0, C08A0, C09A0, C09C0, C09D1, C09D3, C09X0	610406002, 610406009, 610406012, 610406015, 610406028, 610406053, 610406101, 610406157, 610406158, 610406167, 610406168, 610406218, 610406219, 610406284, 610407003, 610407004, 610407005, 610407006, 610407010, 610407011, 610407019, 610407020, 610407021, 610407142, 610407143, 610407144, 610407147, 610407148, 610407151, 610407152, 610407466, 610407467, 610409327, 610409328, 610409331, 610409332, 610412007, 610412013, 610412014, 610412036, 610412037, 610412039, 610412044, 610412045, 610412066, 610412073, 610412080, 610412081, 610412110, 610412129, 610412130, 610412142, 610412143, 610412145, 610412184, 610421318, 610421319, 610421320, 610421321, 610421322, 610421329, 610421330, 610421339, 610421340, 610421346, 610422046, 610422047, 610422063, 610422064, 610422110, 610422111, 610422121, 610422142, 610422163, 610422164, 610422193,

Indication	ATC code	Drug code
		610422194, 610422210, 610422211, 610422212,<br>610422213, 610422214, 610422215, 610422225,<br>610422226, 610422230, 610422231, 610422237,<br>610422327, 610431025, 610431115, 610432011,<br>610432012, 610432013, 610432014, 610432034,<br>610432035, 610433002, 610433007, 610433016,<br>610433017, 610433041, 610433042, 610433066,<br>610433067, 610433068, 610433069, 610433078,<br>610433104, 610443042, 610443043, 610443044,<br>610444017, 610444019, 610444020, 610444022,<br>610444028, 610444030, 610444032, 610444033,<br>610444034, 610444035, 610444062, 610444064,<br>610444065, 610444066, 610444067, 610444073,<br>610444074, 610444075, 610444076, 610444079,<br>610444080, 610444084, 610444085, 610444101,<br>610444119, 610444120, 610444148, 610444153,<br>610444154, 610444158, 610444159, 610444160,<br>610444161, 610444162, 610444163, 610444164,<br>610444165, 610444166, 610444167, 610444169,<br>610444170, 610444172, 610444178, 610453012,<br>610453017, 610453018, 610453025, 610453036,<br>610453037, 610453083, 610453089, 610453090,<br>610453097, 610453121, 610453122, 610453123,<br>610453124, 610453125, 610453126, 610453127,<br>610453147, 610453148, 610453149, 610454035,<br>610454048, 610454080, 610454081, 610461002,<br>610461003, 610461106, 610461108, 610461153,<br>610461204, 610461205, 610462039, 610462040,<br>610463009, 610463010, 610463013, 610463014,<br>610463027, 610463028, 610463039, 610463100,<br>610463125, 610463126, 610463137, 610463138,<br>610463139, 610463140, 610463141, 610463142,<br>610463187, 610463188, 610463211, 610463212,<br>610470001, 610470002, 612120001, 612120004,<br>612120006, 612120016, 612120017, 612120031,

Indication	ATC code	Drug code
		612120054, 612120059, 612120073, 612120119, 612120124, 612120128, 612120148, 612120163, 612120164, 612120165, 612120224, 612120225, 612120226, 612120227, 612120235, 612120252, 612120257, 612120259, 612120266, 612120267, 612120309, 612120317, 612120318, 612120319, 612120320, 612120321, 612120324, 612120330, 612120334, 612120335, 612120336, 612120338, 612120339, 612120341, 612120346, 612120347, 612120348, 612120349, 612120351, 612120356, 612120357, 612130039, 612130043, 612130050, 612130052, 612130071, 612130075, 612130080, 612130086, 612130126, 612130165, 612130169, 612130176, 612130193, 612130198, 612130207, 612130212, 612130217, 612130220, 612130239, 612130249, 612130260, 612130261, 612130267, 612130280, 612130313, 612130314, 612130342, 612130353, 612140073, 612140074, 612140075, 612140128, 612140129, 612140158, 612140159, 612140164, 612140205, 612140206, 612140224, 612140233, 612140234, 612140245, 612140316, 612140327, 612140328, 612140435, 612140436, 612140437, 612140440, 612140443, 612140444, 612140445, 612140450, 612140451, 612140472, 612140479, 612140480, 612140481, 612140483, 612140486, 612140487, 612140489, 612140492, 612140493, 612140494, 612140495, 612140496, 612140497, 612140498, 612140499, 612140500, 612140501, 612140502, 612140503, 612140516, 612140517, 612140518, 612140519, 612140521, 612140526, 612140540, 612140542, 612140545, 612140552, 612140554, 612140555, 612140556, 612140557, 612140558, 612140559, 612140560, 612140561, 612140565, 612140580, 612140590, 612140595, 612140601, 612140614, 612140617,

Indication	ATC code	Drug code
		612140619, 612140620, 612140626, 612140637, 612140647, 612140648, 612140649, 612140650, 612140651, 612140652, 612140655, 612140660, 612140661, 612140662, 612140667, 612140670, 612140671, 612140674, 612140684, 612140685, 612140686, 612140693, 612140694, 612140695, 612140696, 612140697, 612140698, 612140699, 612140700, 612140701, 612140702, 612140703, 612140704, 612140705, 612140706, 612140711, 612140712, 612140713, 612140718, 612140719, 612140720, 612140721, 612140722, 612170015, 612170095, 612170210, 612170340, 612170341, 612170369, 612170457, 612170473, 612170482, 612170485, 612170539, 612170540, 612170547, 612170549, 612170561, 612170594, 612170598, 612170610, 612170631, 612170632, 612170633, 612170647, 612170662, 612170663, 612170664, 612170665, 612170668, 612170669, 612170670, 612170671, 612170672, 612170673, 612170676, 612170677, 612170678, 612170679, 612170680, 612170681, 612170683, 612170692, 612170693, 612170698, 612170699, 612170703, 612170705, 612170706, 612170707, 612170708, 612170709, 612170710, 612190066, 612190103, 612190231, 612190234, 612190236, 612190242, 612190247, 612190250, 620000005, 620000006, 620000040, 620000041, 620000069, 620000075, 620000076, 620000077, 620000080, 620000081, 620000082, 620000126, 620000133, 620000167, 620000168, 620000337, 620000338, 620000339, 620000340, 620001874, 620001875, 620001876, 620001878, 620001881, 620001905, 620001906, 620001954, 620001955, 620001964, 620001990, 620001991, 620001999, 620002000, 620002005, 620002006, 620002007, 620002008, 620002009, 620002010,

Indication	ATC code	Drug code
		620002011, 620002012, 620002013, 620002014, 620002015, 620002016, 620002017, 620002018, 620002019, 620002020, 620002021, 620002024, 620002025, 620002034, 620002035, 620002044, 620002045, 620002048, 620002053, 620002059, 620002060, 620002066, 620002067, 620002069, 620002070, 620002071, 620002072, 620002074, 620002075, 620002078, 620002079, 620002080, 620002083, 620002086, 620002087, 620002132, 620002133, 620002134, 620002135, 620002136, 620002137, 620002138, 620002143, 620002144, 620002145, 620002146, 620002157, 620002158, 620002422, 620002429, 620002430, 620002514, 620002543, 620002607, 620002630, 620002700, 620002701, 620002706, 620002707, 620002708, 620002709, 620002718, 620002719, 620002723, 620002724, 620002732, 620002746, 620002757, 620002758, 620002759, 620002762, 620002819, 620002820, 620002855, 620002856, 620002860, 620002861, 620003083, 620003132, 620003133, 620003178, 620003182, 620003238, 620003262, 620003263, 620003439, 620003440, 620003442, 620003510, 620003512, 620003525, 620003529, 620003560, 620003565, 620003580, 620003581, 620003591, 620003612, 620003617, 620003618, 620003619, 620003620, 620003707, 620003708, 620003709, 620003773, 620003774, 620003807, 620003836, 620003886, 620003887, 620003889, 620003898, 620003899, 620003900, 620003901, 620003902, 620003903, 620003904, 620003906, 620003907, 620003909, 620003951, 620003952, 620003977, 620003978, 620004014, 620004015, 620004016, 620004041, 620004042, 620004043, 620004047, 620004048, 620004049, 620004050, 620004051, 620004052, 620004053, 620004054,

Indication	ATC code	Drug code
		620004055, 620004056, 620004057, 620004058, 620004059, 620004060, 620004061, 620004062, 620004063, 620004064, 620004065, 620004066, 620004182, 620004283, 620004284, 620004286, 620004290, 620004390, 620004414, 620004437, 620004443, 620004453, 620004467, 620004468, 620004477, 620004478, 620004527, 620004539, 620004558, 620004559, 620004581, 620004582, 620004586, 620004593, 620004601, 620004615, 620004617, 620004629, 620004650, 620004654, 620004655, 620004656, 620004758, 620004759, 620004844, 620004907, 620004914, 620004915, 620004919, 620004938, 620004957, 620004966, 620004967, 620004986, 620005002, 620005003, 620005004, 620005006, 620005019, 620005031, 620005049, 620005050, 620005074, 620005075, 620005076, 620005077, 620005097, 620005102, 620005119, 620005192, 620005193, 620005221, 620005243, 620005362, 620005366, 620005429, 620005430, 620005523, 620005524, 620005550, 620005551, 620005552, 620005553, 620005554, 620005555, 620005556, 620005798, 620005824, 620005825, 620005826, 620005854, 620005904, 620005905, 620005927, 620005935, 620005936, 620005953, 620005972, 620006003, 620006004, 620006050, 620006051, 620006106, 620006110, 620006127, 620006128, 620006149, 620006155, 620006158, 620006273, 620006563, 620006564, 620006580, 620006586, 620006597, 620006602, 620006664, 620006665, 620006745, 620006746, 620006793, 620006794, 620006795, 620006796, 620006797, 620006819, 620006820, 620006844, 620006857, 620006879, 620007003, 620007010, 620007014, 620007015, 620007016, 620007070, 620007085, 620007103, 620007146, 620007160,

Indication	ATC code	Drug code
		620007548, 620007817, 620007818, 620007819, 620007820, 620007821, 620007822, 620007823, 620007824, 620007825, 620007826, 620007827, 620007830, 620007831, 620007832, 620007833, 620007834, 620007835, 620007836, 620007837, 620007838, 620007839, 620007840, 620007841, 620007842, 620007843, 620007844, 620007845, 620007846, 620007847, 620007848, 620007849, 620007850, 620007851, 620007852, 620007853, 620007854, 620007855, 620007856, 620007857, 620007858, 620007859, 620007860, 620007861, 620007863, 620007864, 620007865, 620007866, 620007867, 620007868, 620007869, 620007870, 620007871, 620007872, 620007873, 620007874, 620007875, 620007876, 620007877, 620007878, 620007879, 620007880, 620007881, 620007882, 620007883, 620007884, 620007885, 620007886, 620007899, 620007900, 620007917, 620007918, 620007919, 620007920, 620007921, 620007922, 620007923, 620007924, 620007925, 620007926, 620007927, 620007929, 620007930, 620007931, 620007932, 620007933, 620007943, 620007944, 620007997, 620008034, 620008035, 620008036, 620008037, 620008040, 620008041, 620008059, 620008063, 620008064, 620008065, 620008066, 620008067, 620008068, 620008069, 620008281, 620008282, 620008283, 620008285, 620008291, 620008345, 620008347, 620008453, 620008454, 620008455, 620008456, 620008457, 620008458, 620008459, 620008460, 620008461, 620008462, 620008463, 620008464, 620008465, 620008466, 620008467, 620008468, 620008469, 620008470, 620008471, 620008472, 620008516, 620008582, 620008602, 620008603, 620008611, 620008670, 620008677, 620008697, 620008701, 620008715,

Indication	ATC code	Drug code
		620008781, 620008863, 620008864, 620009090, 620009091, 620009092, 620009093, 620009106, 620009118, 620009126, 620009127, 620009199, 620009200, 620009306, 620009307, 620009318, 620009319, 620009358, 620009359, 620009360, 620009363, 620009365, 620009369, 620009370, 620009371, 620009372, 620009373, 620009374, 620009375, 620009376, 620009378, 620009380, 620009383, 620009384, 620009385, 620009386, 620009389, 620009391, 620009392, 620009393, 620009394, 620009395, 620009396, 620009397, 620009400, 620009401, 620009430, 620009434, 620009442, 620009443, 620009453, 620009609, 620009610, 620250701, 620252201, 620252204, 620252207, 620252210, 620252701, 620252712, 620252713, 620253501, 620254406, 620254407, 620254410, 620254411, 620254412, 620254416, 620254419, 620254422, 620254424, 620254509, 620254517, 620254522, 620254527, 620254544, 620254553, 620254554, 620254560, 620255102, 620255302, 620255401, 620255403, 620255408, 620255417, 620255420, 620256101, 620256201, 620262802, 620263001, 620263401, 620263901, 620264701, 620266110, 620266112, 620266119, 620266125, 620266132, 620266138, 620266301, 620266304, 620266305, 620266409, 620266412, 620266413, 620267805, 620268501, 620269701, 620269809, 620270101, 620270703, 620271902, 620272101, 620281201, 620281601, 620281801, 620282601, 620289301, 620289701, 620290102, 620290104, 620290106, 620290111, 620293401, 620294901, 620295101, 620295601, 620295802, 620302701, 620303201, 620303303, 620303501, 620303601, 620303701, 620307001, 620307006, 620307010, 620307012, 620307026, 620307601,

Indication	ATC code	Drug code
		620307701, 620307702, 620309001, 620309101, 620309102, 620318701, 620318801, 620318901, 620319101, 620319704, 620320201, 620320301, 620320401, 620320601, 620323403, 620326701, 620326801, 620327202, 620328101, 620328201, 620328401, 620328602, 620333401, 620333501, 620333601, 620347801, 620348801, 620349101, 620349501, 620350201, 621243302, 621243401, 621243601, 621243701, 621243801, 621244402, 621244501, 621244602, 621245002, 621245201, 621245301, 621245402, 621245501, 621245801, 621246201, 621246301, 621246401, 621246501, 621248601, 621248701, 621248801, 621248803, 621248901, 621249001, 621249102, 621249103, 621365713, 621365810, 621366706, 621366715, 621366718, 621399302, 621399401, 621399602, 621399902, 621400001, 621400301, 621400502, 621400601, 621400901, 621401401, 621401501, 621401701, 621402202, 621402801, 621402901, 621403301, 621469201, 621469301, 621469401, 621469501, 621469601, 621469801, 621470001, 621470101, 621470201, 621470301, 621470401, 621470703, 621470902, 621494801, 621520501, 621526102, 621526202, 621526301, 621530201, 621530501, 621563401, 621573401, 621573402, 621573601, 621575701, 621575702, 621622501, 621622701, 621622801, 621622901, 621623001, 621623101, 621628601, 621628701, 621629301, 621630901, 621631501, 621631601, 621631801, 621631901, 621632201, 621632301, 621632401, 621632501, 621632804, 621632904, 621635101, 621635901, 621636001, 621636401, 621636501, 621636601, 621636701, 621637101, 621638003, 621638103, 621638801, 621638901, 621640901, 621641001, 621641101, 621641601, 621641701,

Indication	ATC code	Drug code
		621642802, 621642902, 621675601, 621675701, 621676601, 621681701, 621681901, 621682001, 621682801, 621682901, 621683601, 621685101, 621685201, 621689701, 621689801, 621720801, 621726901, 621727001, 621733201, 621736902, 621739203, 621739403, 621739702, 621744801, 621751201, 621751301, 621769401, 621769402, 621770001, 621782401, 621783103, 621793502, 621835001, 621835101, 621838103, 621838203, 621838303, 621839502, 621847203, 621847303, 621852302, 621852402, 621852602, 621852702, 621853103, 621853203, 621855203, 621857103, 621859002, 621859102, 621867101, 621890901, 621891001, 621926601, 621926701, 621931301, 621931401, 621931901, 621932001, 621932501, 621934401, 621934402, 621934501, 621934502, 621934601, 621934701, 621936101, 621936801, 621936803, 621936901, 621936903, 621937301, 621937401, 621938501, 621938601, 621941201, 621941301, 621941601, 621941701, 621942301, 621942401, 621942501, 621944301, 621944401, 621945101, 621945201, 621945301, 621945401, 621945501, 621948101, 621948401, 621948501, 621949101, 621949201, 621950402, 621951201, 621951301, 621952601, 621952603, 621952605, 621952701, 621952703, 621952705, 621953901, 621954601, 621954603, 621954701, 621954703, 621956801, 621956901, 621957301, 621957401, 621958501, 621958601, 621958901, 621959001, 621959101, 621959201, 621959301, 621959401, 621959501, 621959601, 621959701, 621959801, 621960401, 621960402, 621965001, 621965002, 621965401, 621965402, 621966001, 621966002, 621966101, 621966201, 621966202, 621966301, 621966302, 621973001, 621973101, 621974401,

Indication	ATC code	Drug code
		621974402, 621974501, 621974601, 621975302, 621977100, 621977101, 621977601, 621977602, 621980801, 621982301, 621985102, 621985202, 621985501, 621985601, 621986501, 621986601, 621987002, 621991101, 621991201, 621992201, 621992601, 621999101, 622002701, 622002801, 622006801, 622007201, 622010101, 622010502, 622015601, 622018401, 622018501, 622022201, 622022301, 622023701, 622023901, 622024201, 622024301, 622025401, 622025404, 622025501, 622025504, 622029401, 622029402, 622029501, 622029601, 622031901, 622035901, 622035902, 622036401, 622038701, 622039201, 622039202, 622039301, 622039302, 622039401, 622049001, 622049101, 622049501, 622049601, 622049701, 622050101, 622050201, 622050301, 622052101, 622052102, 622055501, 622055502, 622058601, 622060401, 622060701, 622060702, 622062801, 622062802, 622063901, 622064001, 622064002, 622064401, 622064403, 622064801, 622064802, 622065501, 622065601, 622065701, 622065801, 622067201, 622068001, 622068002, 622071301, 622071401, 622072601, 622076101, 622076901, 622077601, 622094701, 622095501, 622095601, 622097101, 622098601, 622098701, 622099401, 622099501, 622100201, 622100202, 622101901, 622102201, 622102301, 622108001, 622108801, 622108802, 622109301, 622110101, 622110102, 622110301, 622117301, 622117401, 622125301, 622125302, 622125401, 622125402, 622128401, 622128402, 622128501, 622128502, 622134301, 622134401, 622134501, 622136701, 622136801, 622136901, 622137601, 622137901, 622138901, 622139001, 622139101, 622140101, 622140201, 622140301, 622142401, 622142501, 622142502,

Indication	ATC code	Drug code
		622142601, 622142701, 622142801, 622143001, 622143101, 622143501, 622143601, 622144801, 622144901, 622145001, 622145901, 622146001, 622146401, 622146501, 622146601, 622146801, 622146901, 622147001, 622150101, 622150201, 622151701, 622151801, 622152401, 622152501, 622153001, 622153101, 622153201, 622153501, 622153601, 622153701, 622155001, 622155101, 622155201, 622156601, 622156701, 622158001, 622158101, 622158201, 622160301, 622160401, 622160501, 622161501, 622161502, 622162201, 622162301, 622162401, 622163001, 622163101, 622163201, 622163901, 622165301, 622165401, 622165501, 622166501, 622166601, 622166701, 622168601, 622168701, 622168801, 622171001, 622171101, 622171201, 622172301, 622172401, 622172501, 622173501, 622173601, 622173701, 622174301, 622174601, 622174701, 622174801, 622176001, 622176101, 622177001, 622177101, 622177201, 622180801, 622181201, 622181301, 622181401, 622181901, 622182001, 622183801, 622184701, 622184801, 622187801, 622187901, 622189801, 622189901, 622193201, 622198101, 622199201, 622199301, 622201301, 622201401, 622202301, 622202901, 622206801, 622206901, 622207401, 622207501, 622208801, 622213801, 622214101, 622214201, 622214301, 622215001, 622216201, 622216301, 622219501, 622219601, 622221101, 622221501, 622224601, 622230401, 622230501, 622233401, 622233501, 622233601, 622236201, 622236301, 622239501, 622240001, 622240101, 622241101, 622241201, 622242401, 622243401, 622243501, 622243801, 622247001, 622247101, 622249501, 622249601, 622249801, 622251401, 622251501, 622253701, 622253801,

Indication	ATC code	Drug code
		622253901, 622255401, 622255501, 622255601, 622255701, 622257701, 622257801, 622257901, 622258201, 622258301, 622260701, 622260901, 622261701, 622264101, 622264201, 622267201, 622267301, 622269701, 622271001, 622272201, 622272301, 622272401, 622273501, 622279501, 622280101, 622281901, 622282601, 622284001, 622284101, 622284601, 622284801, 622285601, 622287301, 622288501, 622289401, 622296701, 622298501, 622301601, 622301901, 622302001, 622302101, 622307501, 622307601, 622307701, 622308701, 622308801, 622309101, 622310500, 622312800, 622315800, 622318700, 622319800, 622320700, 622323000, 622324500, 622333601, 622333701, 622333801, 622333901, 622334001, 622334401, 622334501, 622334601, 622334701, 622334901, 622335001, 622335101, 622335201, 622335501, 622336901, 622337001, 622337101, 622337401, 622337501, 622337601, 622337701, 622337901, 622338801, 622338901, 622339001, 622339101, 622339201, 622339301, 622339401, 622340401, 622340501, 622340601, 622340701, 622340801, 622341401, 622341701, 622341801, 622342201, 622342301, 622343001, 622343201, 622343301, 622343401, 622343501, 622343601, 622343701, 622343801, 622343901, 622344401, 622345701, 622345801, 622345901, 622346001, 622346101, 622346401, 622346501, 622346601, 622346701, 622346801, 622347301, 622347601, 622347701, 622347801, 622348001, 622348101, 622348201, 622348301, 622348401, 622348601, 622348801, 622348901, 622349001, 622349101, 622349301, 622349801, 622349901, 622350001, 622350101, 622350801, 622350901, 622351001, 622351201, 622351501, 622351601, 622351701,

Indication	ATC code	Drug code
		622351801, 622351901, 622352401, 622352501, 622352601, 622352701, 622352801, 622353201, 622353301, 622353401, 622353501, 622353601, 622353701, 622353801, 622354101, 622354201, 622355001, 622355101, 622355201, 622355301, 622355501, 622355601, 622355701, 622355801, 622355901, 622356001, 622356101, 622356701, 622356901, 622357001, 622357101, 622357201, 622357301, 622357401, 622357501, 622357801, 622357901, 622358001, 622358101, 622358601, 622358701, 622358801, 622358901, 622359001, 622359401, 622359501, 622359601, 622359701, 622359801, 622360701, 622360801, 622362101, 622365401, 622365501, 622365601, 622365701, 622367401, 622367501, 622367601, 622367701, 622368501, 622368601, 622369201, 622369301, 622369401, 622369501, 622369601, 622369701, 622369801, 622369901, 622370501, 622370601, 622371301, 622371401, 622371501, 622371601, 622372001, 622372101, 622372201, 622372301, 622373401, 622373501, 622373601, 622373701, 622376601, 622376701, 622377201, 622377401, 622377501, 622377601, 622377701, 622378401, 622378501, 622378601, 622379601, 622379701, 622379801, 622379901, 622380601, 622380901, 622381001, 622381501, 622381601, 622381701, 622381801, 622382201, 622382301, 622382401, 622382501, 622382601, 622382701, 622382801, 622382901, 622383401, 622383701, 622383801, 622383901, 622385001, 622385101, 622385201, 622385301, 622385601, 622386001, 622386401, 622386501, 622386601, 622386701, 622387101, 622387201, 622388301, 622388401, 622390201, 622390301, 622390401, 622390501, 622390601, 622390701, 622390801, 622390901, 622391301,

Indication	ATC code	Drug code
		622391401, 622391501, 622391601, 622392601, 622392701, 622392801, 622392901, 622393501, 622394201, 622394301, 622395401, 622395501, 622395601, 622395701, 622396101, 622396201, 622396301, 622396401, 622397701, 622397901, 622398001, 622398101, 622398201, 622398301, 622398501, 622398901, 622399101, 622399301, 622399501, 622399601, 622399701, 622399901, 622412201, 622412301, 622422901, 640407031, 640432013, 640433003, 640433004, 640433068, 640453076, 640453077, 640462031, 640462042, 642120006, 642130005, 642130006, 642130015, 642130019, 642140004, 642140005, 642140006, 642140021, 642140022, 642140023, 642140024, 642140026, 642140027, 642140030, 642170018, 642170019

Supplementary Table S4. Procedure codes of dialysis used in the case study.

Procedure name	Procedure code
Dialysis	113002510, 114003510, 114003610, 114006610, 114008250, 114009310, 114009410, 140007710, 140008170, 140008510, 140008770, 140008810, 140029850, 140033770, 140036710, 140051010, 140051110, 140052810, 140054850, 140054950, 150148610, 150148750, 150148850, 150151150, 150151250, 150152710, 150322910, 190167970

S4. Separation experiment of healthcare insurance claims of twin children

We performed a trial to exploit machine learning techniques to separate healthcare insurance claims of same-sex twin children. Specifically, we constructed classifier models and applied the state-of-the-art learning algorithms (gradient boosting decision tree, multilayer perception, and support vector machine) to learn the contents of claims, such as the number of claims and the cardinality of diagnosis codes. We then validated the accuracy of classification by using the Mie and Gifu datasets. Supplementary Table S5 summarizes the result, suggesting that the trial is not successful at present.

Supplementary Table S5. The accuracy of classifier models that distinguish patients who have the same ID1 value by utilizing the state-of-art machine learning algorithms (GBDT, gradient boosting decision tree; MLP, multilayer perceptron; and SVM, support vector machine) and the feature variables generated from the claims. The feature variables include the following: (a) gender and age; (b) the number of claims; (c) cardinality of disease diagnosis codes, medical treatment codes, drug prescription codes, and medical institution codes; and (d) the number of months of visits to a medical institution. The models were trained and validated using prefecture-level claims datasets (Mie and Gifu datasets) in terms of AUC, area under the ROC curve; F-measure, the harmonic mean of Precision and Recall.

**Training dataset**	**Mie**			**Gifu**		
**Validation dataset**	**Gifu**			**Mie**		
**Learning algorithm**	**GBDT**	**MLP**	**SVM**	**GBDT**	**MLP**	**SVM**
**AUC**	0.951	0.945	0.949	0.938	0.937	0.936
**Precision**	0.005	0.005	0.006	0.005	0.005	0.005
**Recall**	0.927	0.894	0.915	0.908	0.906	0.912
**F-measure**	0.011	0.010	0.011	0.010	0.010	0.010
